# Supplementary material for: Large-scale sequence comparisons with sourmash
Source: F1000Res. 2019 Jul 4;8:1006. [Version 1] doi: 10.12688/f1000research.19675.1 (PMC6720031; doi:10.12688/f1000research.19675.1)
Supplement: Peer review history

# Peer review: Reviewer response for version 1

**DOI:** 10.5256/f1000research.21579.r52218

**Published:** 2019-09-02

**Authors:** Rayan Chikhi

**Affiliations:** 1 C3BI USR 3756, CNRS (French National Center for Scientific Research), Institut Pasteur, Paris, France

The authors present sourmash 2, a tool that implements a novel combination of SBTs and MinHashes, which are both fascinating computational concepts; thus, their mix is quite an interesting one. Sourmash 2 enables to perform large-scale sequences-vs-database similarity searches. The article offers a comprehensive guide for many of the software features, with biologically relevant scenarios. This is a useful contribution that is highly relevant to current needs in biology. There are a few technical issues with the current manuscript version that I list below. But otherwise, most of my remarks are for adding some extra perspective. I believe the manuscript can be approved after the technical fixes.

 Major remarks:

1. A quick recap of the state of the art in containment search would be helpful. Here you claim to use ‘a modulo approach’. Mash screen and containment minhash use different approaches (see e.g. the blog post of ‘Mash screen’). It would be nice if, in this paper, the usage of the modulo approach was put into perspective compared to those two aforementioned methods.
2. In fact, in the blog post cited as reference 8, Ondov writes that “the modulo approach is problematic for metagenomic applications (e.g. finding a virus in a metagenome).” The problem is indirectly mentioned in the manuscript (“can sacrifice some of the memory and storage benefits of standard MinHash techniques, as the signature size scales with the number of unique k-mers”). It would be neat to get the authors’ comparative perspective here as to why using modulo is the better approach.
3. My main comment would perhaps be the lack of comparison with other software. I do not know if this is a requirement for F1000Research in the “Software Tool Article” category. I suppose that sourmash is the only tool that implements SBT-Minhashes, so of course here there is no competitor in that category. It would however be nice to have some indication on whether sourmash is best-in-class in each of the proposed features (the uses cases), or whether other tools already exist and somehow do a similar job. And, the other way around, which areas where sourmash is really the only tool capable of doing X in reasonable time. I do not expect a comprehensive benchmark, but some informal indication would already be appreciated.
4. What are roughly the limits of similarity queries? E.g. sequences shorter than X or having identity below Y% have no chances to be reported.
5. A summary of all the features demonstrated in the main text could be helpful. For instance, reading only the introduction, it is not explicit that a natural application of sourmash is outlier/contaminant detection.

Minor remarks:

1. “Sequence Read Archive now contains over 20 Petabases of data1”: seems to be over 30 PB in 2019 according to the plot in reference 1.
2. It is not clear what the ‘LCA’ term stands for in the context of the database format introduced here. Is it the lowest common ancestor?
3. The description of LCA (in section “LCA database”) is imprecise. What does a “named list” mean in this context? A Figure would be helpful to see a small example.
4. A sentence in the manuscript mentions ‘a second database format’. The ‘first format’ is supposedly the SBTMH but it is only implicit that SBTMH is a ‘format’.
5. The introduction mentions a bunch of features implemented in other tools (“Jaccard similarity comparisons, ..,  k-mer abundance comparisons, decrease runtime and memory requirements, and work on streaming input data.”) Are all of these implemented in sourmash2, or only a subset of them? (It seems to me that most are implemented.)
6. The description of the modulo approach used is imprecise. How is the hash space divided into s equal ‘bands’ (undefined term), precisely? Also, I suppose this somewhat different from the modulo approach proposed by Broder, and clarified in Mash screen’s blog post, but how so?
7. The concept of ‘hash subset retention’ is not well defined. I suppose it is the set of hashes that result from a MinHash computation.
8. Abundance filtering (as in Finch) is not performed in sourmash2, right?
9. Some of the ‘signature utilities’ are self-explanatory. However, what is the difference between ‘intersect’ and ‘overlap’? What is ‘flatten’?
10. Regarding the sentence: “although we recommend using more accurate approaches for detailed comparisons.” To make the paper self-contained, a short explanation would be needed to delineate what sort of concrete use-case(s) is/are meant behind the term ‘detailed comparisons’.
11. The “similarity queries” and “containment queries” sections could benefit from at least one use-case example per query. This is to illustrate the two sections, which are a bit obscure without examples. (I realize that use cases are given later in the codes examples, so perhaps a forward-reference could work, albeit less elegant.) A proposal for similarity queries: ‘find all genomes in the SBTMH (leaves) that are similar to a query genome’.
12. Awkward formulation of that part of the sentence:  [..] ‘using a k-mer size of 4, use all 4-mers, and track abundance.’ (although I understood that the k-mer size was 4 and the ‘use all k-mers’ refers to a scaling factor of 1)
13. The “Tetranucleotide Frequency Clustering“ section is quite nice. It should be emphasized however this isn’t really a minhash sketch: all 4-mers are considered.
14. Regarding the sentence “We see that the minimum similarity in the matrix is 0%”, how is that seen? visual inspection of ecoli.comp.matrix.png?
15. Regarding the sentence “This process is repeated until the threshold is reached.”: I forgot.. which threshold?
16. Regarding the sentence “We see that 20.1% of k-mers match 82 genomes in GenBank.”: how is this seen? Also “we see two matches between P. ruminis strains among all matches.” In the output above that text, I see only one match. (I could not test that section due to the missing download URL.)

Regarding the software commands:

- As an important note, one cannot easily copy-paste the command lines as short dashes (-) are converted to long dashes (–). Nevertheless, I still automatically replaced all the dashes and tested all command lines. I’ll report any problem below.
- Extra ‘\’ at the end of the command: “curl –L –o ecoli–reads.khmer.fq.gz     https://osf.io/26xm9/download \”.
- Missing url in command (and also extra ‘\’ at the end): “[..] unmapped–qc–to–ref.fq.sig https://osf.io//download \”   Thus I could not test this part.
- The streaming operation at the beginning of the MDS section overwrites the file ‘ERR458584.khmer.sig‘ produced before, perhaps make a note of that.
- The url “curl –L –o genbank–d2–k31.tar.gz \
       https:// s3–us–west–2.amazonaws.com/
       sourmash–databases/2018–03–29/
       genbank–d2–k31.tar.gz“ has extra new lines
- In the command “sourmash index –k 31 ecolidb \
       escherichia–sigs /*.sig”, a space is wrongly inserted after “escherichia-sigs”
- And also, in the command that follows, one of the ‘\’ is extra and another ‘\’ is missing.
- The SBT path inside the file ‘fungi-k31.sbt.json’ is wrongly hardcoded. Also, when fixing it, I get “WARNING: this is an old index version, please run `sourmash migrate` to update it.” Although it did end up working.

I confirm that I have read this submission and believe that I have an appropriate level of expertise to confirm that it is of an acceptable scientific standard.

# Peer review: Reviewer response for version 1

**DOI:** 10.5256/f1000research.21579.r52588

**Published:** 2019-08-27

**Authors:** Brad Solomon

**Affiliations:** 1 Department of Computer Science, Whiting School of Engineering, Johns Hopkins University, Baltimore, MD, USA

This manuscript presents an improved software library, sourmash 2.0, for the efficient construction and analysis of MinHash sketches of genomic and proteomic sequence data. The primary innovations of sourmash 2.0 are the novel applications and modified implementations of several existing sketching and indexing methods. In particular, the two main advancements are (1) a ‘modulo approach’ to sketch construction and the development of a modified Sequence Bloom Tree (SBT) index over MinHash sketches. In addition, unlike conventional MinHash methods, sourmash 2.0 can also track the abundance of retained hash elements, allowing some degree of abundance comparison and abundance estimation of genomic datasets. 

 Multiple use cases for sourmash 2.0 are provided including sketch construction, similarity comparisons, containment querying, classification, and metagenome decomposition. The manuscript is very thorough in describing sketch construction with example run commands for full-length genomes, proteomes, as well as raw reads. The ease of use is further demonstrated with a single line command that, while involving multiple pipes, is capable of constructing a signature through a download data stream. Similarly, several uses of sketch comparisons are demonstrated including visualization and outlier detection. Lastly the use of the SBT MinHash (SBTMH) is demonstrated for classification or containment of arbitrary sequence queries. The download instructions and example run commands are fully functional and the input datasets are reasonably sized for toy examples (most on the order of <100 MB).

 Excluding the description of the modulo approach of sketch construction, the manuscript itself is technically sound. The topic is high impact -- sketching approaches are increasingly popular solutions to a multitude of research topics in computational biology. Many of these potential use cases are demonstrated successfully in the manuscript. That said, there are numerous existing solutions to each of the use cases presented in the manuscript and little to no attempt was made to provide benchmarking information or to demonstrate the improvements sourmash 2.0 has over its competitors. While sourmash 2.0 has an ease of use that will undoubtedly facilitate use, it does not adequately demonstrate an improved capacity for analysis over these other tools. This is primarily a suggestion for improvement as, based on the F1000 Research guidelines, the manuscript is correct and valid in its current state.

 Major Comments:

1. The ‘modulo approach’ for sketch construction, despite being one of the main innovations of the method, is particularly unclear in the manuscript. The cited literature (Broder 1997) describes an approach that sub-samples hash values based on a modulo factor to address the inherent weakness of a Minhash in a mixture of several distinct components. However the description of the sourmash implementation instead describes splitting the hash space into ‘equal bands’ and selecting only the minimum band. As the existing modulo approach has no guarantees on equal-sized (or even equal-fraction as the manuscript claims elsewhere) sub-sampling, this appears to be a novel and significant contribution to the field. However there are no details that explain (1) how the hash space is divided, (2) how the minimum band is selected, and (3) how downsampling is performed. 
2. Sourmash 2.0 is motivated by “a particular focus towards enabling efficient containment queries using large databases”. However the manuscript does not include any true comparisons about sourmash’s performance against existing tools, alternative approaches, or benchmarking information for even conventionally sized datasets. This greatly limits the potential impact of sourmash given there are many competing sketch strategies and an even larger range of available implementations.
  While it is unreasonable to expect a full review of the available methods, the inclusion of even a single ‘large-scale’ dataset in the test set or use cases would go a long way towards demonstrating the scalability of sourmash. Selecting a biologically relevant subset from a public genomic repository such as the NIH SRA, TCGA, or GTEx (to name just a few) would alleviate the need to host such a dataset while allowing large-scale reproducibility and benchmarking.

Minor Comments:

1. The ‘Salmon & edgeR’ MDS plot in Figure 1 does not have points associated with the text labels. As there is no consistency in the placement of labels versus nodes in the first plot (Sourmash Compare MDS), even approximate values are difficult to determine. 
2. The commands listed in the manuscript are using the wrong character (‘–’ vs ‘-’). None of them run properly without manual adjustments or retyping. I imagine this is a formatting issue more than a coding one but it would make reproducing the results a lot simpler if it was resolved.

Other: 

1. The inclusion of limited abundance information is a particularly interesting improvement over standard MinHash sketches. The manuscript suggests that the abundance tracking can play a significant role when ‘comparing many signatures’ but there is no concrete claim to assess. While outside the scope or focus of this work, a follow-up piece which explores the theoretical or practical impact of systematically sub-sampled counting information would be potentially high impact. 

I confirm that I have read this submission and believe that I have an appropriate level of expertise to confirm that it is of an acceptable scientific standard.
